# Genetic Generalized Epilepsy and Intrafamilial Phenotypic Variability with Distal 7q11.23 Deletion

**DOI:** 10.1177/2329048X221093173

**Published:** 2022-04-21

**Authors:** Veronica Birca, Kenneth A. Myers

**Affiliations:** 1 Division of Child Neurology, Department of Pediatrics, McGill University, Montreal, Quebec, Canada; 2 Research Institute of the McGill University Health Centre, Montreal, Canada; 3 Department of Neurology & Neurosurgery, Montreal Children’s Hospital, McGill University, Montreal, Canada

**Keywords:** 7q11, copy number variant, epilepsy

## Abstract

**Background:** Distal 7q11.23 deletions are variably associated with epilepsy, intellectual disability and neurobehavioural abnormalities. The relative importance of different genes in this region in contributing to different phenotypes is not clear, though *HIP1* and *YWHAG* are both thought to play important roles. **Patients and Methods:** We performed thorough phenotyping on members of a family in which multiple members carried a relatively small 0.8 Mb distal 7q11.23 deletion, affecting 17 genes. **Results:** Two brothers and a half-brother had all inherited the 7q11.23 deletion from their mother. The eldest two both had global developmental impairment and genetic generalized epilepsy, involving absence, myoclonic or myoclonic-atonic seizures. There was no history of seizures in the mother or her youngest son, but both also had developmental impairment. **Conclusion:** Distal 7q11.23 deletions affecting *HIP1* and *YWHAG* may cause developmental impairment and genetic generalized epilepsy, with considerable intrafamilial phenotypic variability.

## Background

Distal microdeletions of chromosome 7q11.23 have been associated with some or all of: epilepsy, intellectual disability, learning difficulties and neurobehavioral abnormalities.^1^ These microdeletions are adjacent to the common Williams-Beuren syndrome deletion region on chromosome 7q11.23.^2^ The two genes commonly deleted are *HIP1* (OMIM 601767) and *YWHAG* (OMIM 605356), encoding huntingtin-interacting protein 1 and tyrosine monooxygenase-3/tryptophan 5-monooxygenase activation protein, gamma isoform, respectively.^1^

The vast majority of published cases have been single patients, so it is unclear to what degree phenotypic variation is related to differences in deletion size or variable penetrance. We describe a family in which multiple individuals with the same 7q11.23 microdeletion had markedly different clinical features, illustrating that penetrance of this copy number variant can be quite variable. We also review previously published cases and describe the typical epilepsy phenotypes that may be associated.

## Patients and Methods

**Figure 1.**
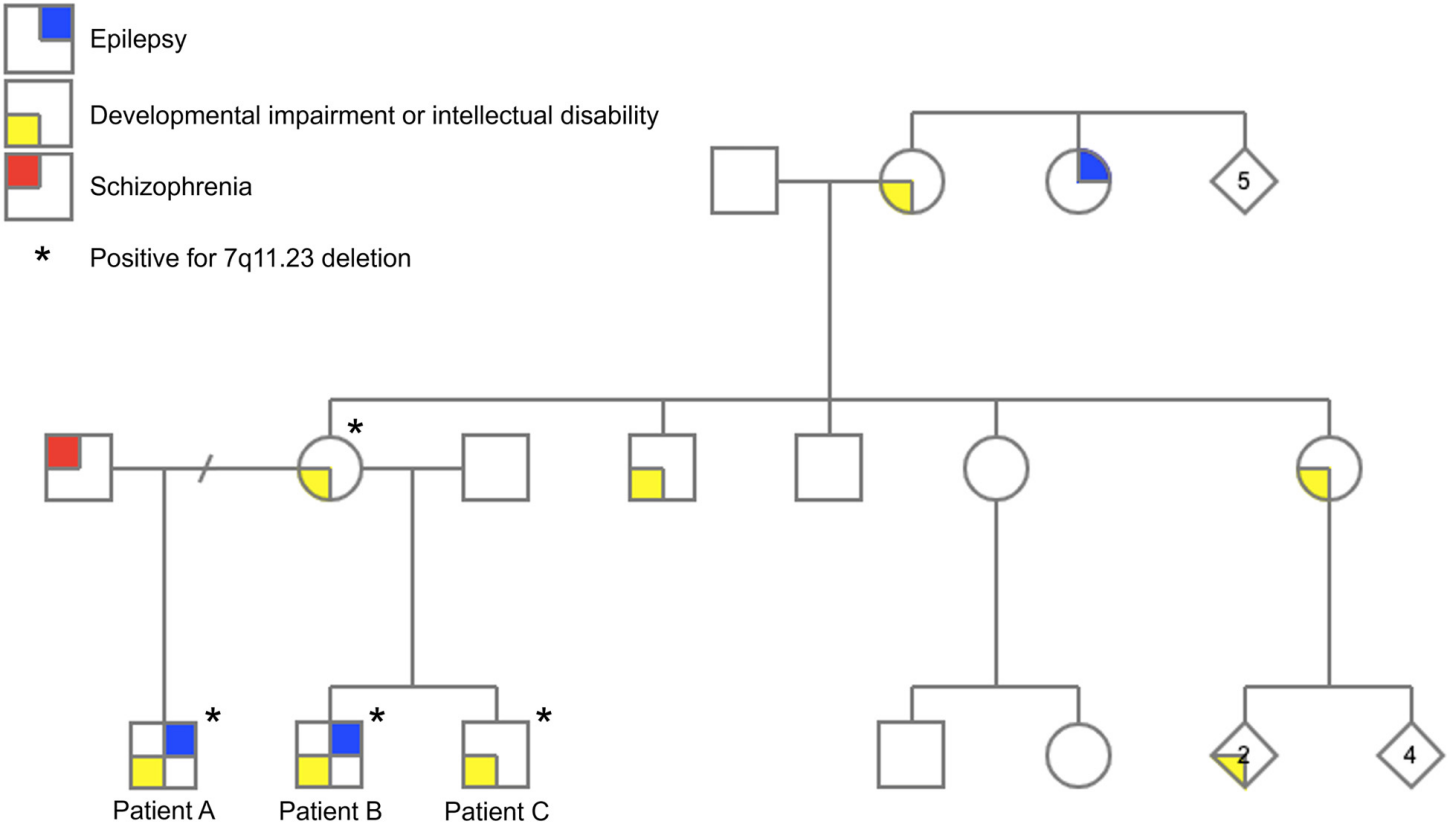
Pedigree of a family with 7q11.23 deletion.

**Figure 2.**
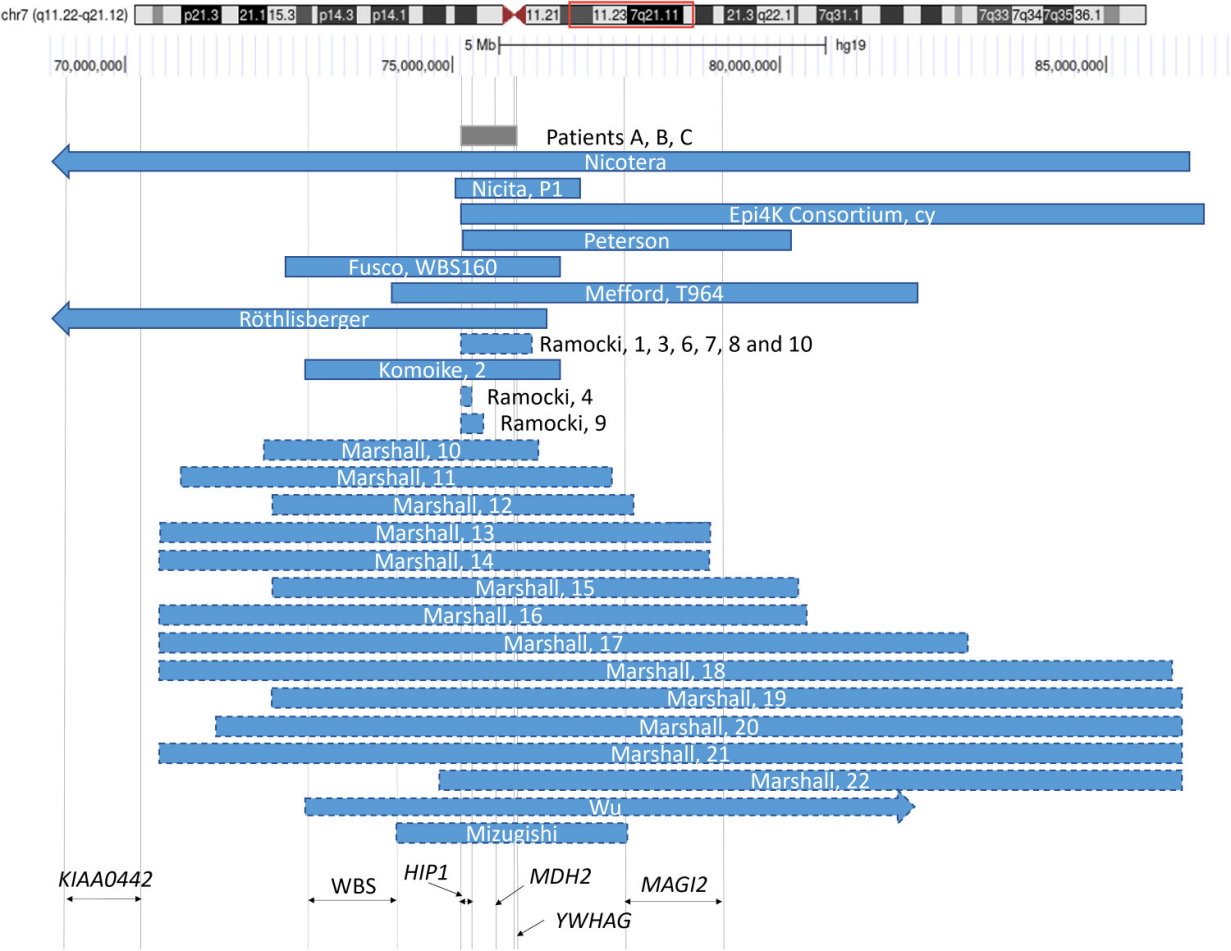
Deletions including 7q11.23 in patients with epilepsy. Deletions are shown in decreasing order of year of publication. Patients A/B/C are reported in the current publication; individuals identified through literature review are identified with the author name and patient ID in the original publications. The deletion of patients A/B/C affects 17 genes: *HIP1*, *CCL26*, *CCL24*, *RHBDD2*, *POR*, *MIR4651*, *SNORA14A*, *TMEM120A*, *STYXL1*, *MDH2*, *SRRM3*, *BC063788*, *HSPB1*, *AX747594*, *YWHAG*, *SSC4D*, and *ZP3*. Dashed contours indicate an estimated breakpoint from figures in original publications, or provided information on deleted genes, since precise coordinates were not given by the authors. Dotted portions indicate a range of breakpoint, since the precise breakpoint was undefined in the original publications. When the original human genome assembly was specified, breakpoints were remapped to the GRCh37/hg19 assembly to facilitate a more accurate comparison with the patients reported in this study, using the National Center for Biotechnology Information (NCBI) Genome Remapping Service.^3,4^ Figure includes a screenshot from UCSC genome browser (http://genome.ucsc.edu). Abbreviations: WBS = Williams-Beuren syndrome common deletion.

A boy aged 14 years (patient A), and his two maternal half-brothers aged eight years (patient B) and four years (patient C), had epilepsy, developmental impairment, learning difficulties and/or neurobehavioral abnormalities (Figure 1). All shared with their mother the same heterozygous microdeletion in cytogenetic band 7q11.23, at position chr7:75163662–76047662 (hg19), of approximately 0.8 Mb, encompassing 17 genes (Figure 2). Informed written consent was obtained from the patients’ caregivers; the study was approved by the McGill University Health Centre Research Ethics Board (2018-3937).

## Results

The family was non-consanguineous, of French-Canadian and Acadian background. The patients’ mother had developmental impairment and learning difficulties, but no history of seizures. There was an extensive history of developmental impairment with or without epilepsy, in multiple family members on the maternal side (Figure 1). The paternal family history for patient C could not be obtained. The two fathers did not undergo chromosomal microarray analysis. There was no reported epilepsy, developmental impairment/intellectual disability in the paternal family history for patients A and B. A more complete pedigree and extension of the genetic testing to other family members was not possible due to the patients being lost to follow-up.

### Patient A

The eldest boy had epilepsy with myoclonic-atonic seizures and moderate intellectual disability (Tables 1 and 2). Seizures began at 4 years of age and initially involved sudden loss of awareness and staring, with subtle myoclonic jerks, corresponding to absence seizures on video-EEG. At age 6 years, parents described episodes with prominent jerks. Stereotyped episodes were captured on video-EEG, showing maximally bifrontal generalized bursts of 3 Hz spike-wave or polyspike-wave associated with behavioral arrest, head/upper extremities jerks and head loss of tone, corresponding to myoclonic-atonic seizures. Epileptiform activity was enhanced with photic stimulation and hyperventilation. The background tracing was otherwise normal.

**Table 1.** Epilepsy Features in Patients with Distal 7q11.23 Deletions and Epilepsy.

Publication author, patient ID	Sex/Age	Sz onset	Sz types (initial sz type in bold)	EEG	Epilepsy phenotype	Anti-seizure medications (ineffective in italics) and epilepsy course
A (current publication)	M/14 year	4 year	**Absence**, myoclonic	Generalized 3-5 Hz SW and PSW + /- myoclonic jerks, with photoconvulsive response; bifrontal 3-5 Hz SW or PSW without clinical correlate, enhanced during hyperventilation	Epilepsy with myoclonic-atonic sz	ESM, *LEV* (early discontinuation due to aggressivity, that did not resolve after discontinuation), LTG, *TPM* (early discontinuation due to decreased alertness and sleepiness), VPA being increased (seizure recurrence with attempt to wean off)
B (current publication)	M/8 year	2.5 year	Absence, myoclonic	Generalized spike and PSW + /- myoclonic jerk, R parietal SW induced by hyperventilation without clinical correlation	GGE	VPA progressively increased, pending EEG to rule out uncontrolled absence seizures
C (current publication)	M/4 year	18 months	Possible absence sz	Normal	—	None
Nicotera, -	F/13 year	Neonatal period	**Neonatal seizures**, IS, followed by variable sz types including focal, FBTC, or flexion-extension spasms associated with vital sign changes	Quasi-continuous frontocentral SW (age 13 y)	West syndrome	ACTH, cannabidiol, CLB, CZP, *ESM*, hydrocortisone*, LCM*, LEV, *LTG*, *NZP*, *OXC*, *PER*, PGB, PB, *RFN*, TPM, *VGB, VPA*; Drug-resistant with significant improvement with cannabidiol
Nicita, P1	M/3 year 10 months	1 year 2 months	**Focal motor, FBTC**	R temporo-occipital SW with diffusion	—	CBZ (partial reduction), LEV (seizure-freedom)
Epi4 K Consortium, cy	−/−	3 months	**IS**, atypical absence	Hypsarrhythmia with superimposed multifocal independent SW	—	—
Peterson, -	F/21 year	≤12 year	-	—	—	VPA; worsening despite daily medicationss upon re-evaluation at 20 y
Fusco, WBS160	F/14.6 year	2 year	GTC	—	Epilepsy with GTC sz alone	LTG
Mefford, T964	F/20 year	9 months	**FS**, generalized sz (GTC, myoclonic, and atypical absence)	Fast and slow GSW, PSW	Generalized	CLB, DZP, ESM, LTG, PB, VPA
Röthlisberger	F/15 months	3 months	IS	Hypsarrhythmia	West	Drug-resistant
Ramocki, 1	F/1 mo	Neonatal period	—	—	—	— Death in neonatal period
Ramocki, 1′s father	M/34 year		Generalized		Generalized	
Ramocki, 3	M/4 year	—	FS, generalized	—	Generalized	—
Ramocki, 4	F/13 year	—	Generalized	—	Generalized	Drug-resistant, VNS placement
Ramocki, 4's brother	M/14 year	—	Generalized	—	Generalized	—
Ramocki, 6	F/4 year	—	Focal and generalized	—	Mixed	—
Ramocki, 7	F/9 year	—	Focal	—	Focal	Drug-resistant
Ramocki, 7's sister	F/11 year	—	Focal	—	Focal	—
Ramocki, 7′s father	M/38 year	—	Focal	—	Focal	—
Ramocki, 8	F/13 year	—	Generalized	—	Generalized	—
Ramocki, 9	M/3 year	—	Focal	—	Focal	—
Ramocki, 10	M/19 year	—	Generalized	—	Generalized	—
Komoike, 2	F/-	18 mo	IS	Hypsarrhythmia	West syndrome	*VPA, ZNS, CBZ*, ACTH
Marshall, 10	M/-	—	IS	Variation form of hypsarrhythmia	West syndrome	—
Marshall, 11	F/−	4 months	IS	Hypsarrhythmia	West syndrome	—
Marshall, 12	M/−	—	IS, myoclonic, tonic	—	—	—
Marshall, 13	M/−	—	IS	—	—	—
Marshall, 14	F/−	—	IS	—	—	—
Marshall, 15	M/−	5 months	IS, focal	—	—	—
Marshall, 16	M/−	—	IS	—	—	—
Marshall, 17	M/−	2 months	IS	Hypsarrhythmia	West syndrome	—
Marshall, 19	F/−	—	Absence	—	—	—
Marshall, 20	F/−	—	IS	—	—	—
Marshall, 21	M/−	—	**IS**, followed by sz during childhood	—	—	—
Marshall, 22	M/−	—	Myoclonic	—	—	—
Wu	F/6.5 year	—	Absence	—	—	—
Mizugishi	M/4 year		IS	Hypsarrhythmia	West syndrome	VPA, NZP, ACTH; Drug-resistant

Abbreviations: FBTC = focal-to-bilateral-tonic-clonic, FS = febrile seizure(s); GGE = genetic generalized epilepsy; GSW = generalized spike-wave; GTC = generalized tonic-clonic; IS = infantile spasms; Mo = month(s); PSW = polyspike-wave; R = right; SW = spike-wave(s); Sz = seizure(s); VNS = vagal nerve stimulator; Y = years.

Anti-seizure medication abbreviations: CBZ = carbamazepine; CLB = clobazam; CZP = clonazepam; DZP = diazepam; ESM = ethosuximide; LCM = lacosamide; LEV = levetiracetam; LTG = lamotrigine; NZP = nitrazepam; OXC = oxcarbazepine; PB = phenobarbitone; PER = perampanel; PGB = pregabalin; RFN = rufinamide; TPM = topiramate; VGB = vigabatrin; VPA = valproate; ZNS = zonisamide.

**Table 2.** Developmental Impairment and Other Clinical Features in Patients With Distal 7q11.23 Deletions and Epilepsy.

Publication author, patient ID	Sex/Age	Early developmental milestones (sat/walked/first word); ID degree or developmental impairment	Neurobehavioral or neuropsychiatric abnormalities	Micro/macrocephaly / MRI brain	Dysmorphic features or systemic illnesses
A (current publication)	M/14 year	-/12 mo/11 mo; Moderate, learning difficulties	ADHD	No / Normal (age 5)	Dolichocephaly, low-set ears and telecanthus
B (current publication)	M/8 year	9 mo/16-18 mo/16-18 mo; GDD (fine motor, language)	ADHD	No / -	—
C (current publication)	M/4 year	6 mo/12 mo/9 mo; GDD (fine motor, language)	Oppositional behavior, wakes up 5-6 times each night	No / -	Alternating exotropia
Nicotera, -	F/13 year	-/not walking/no words; Severe, born at 32 weeks of gestational age	—	Microcephaly / Thinning of corpus callosum, reduced brainstem size, thinning of periventricular white matter, bilateral optic nerve hypoplasia (age 11 y)	Depressed nasal bridge, broad nose, wide and prominent open mouth, puffiness around eyes and lips, full cheeks; failure to thrive, recurrent kidney stones, scoliosis
Nicita, P1	M/3 year 10 months	-; Mild, language delay	Hyperactivity	- / Normal	—
Epi4 K Consortium, cy	−/−	-; Severe, few words at 5 year, cognitive functioning at level of 1-2 year	—	- / Mild prominence of third and fourth ventricles, mild thinning of normal corpus callosum	—
Peterson, -	F/21 year	8 mo/by 16 mo/-; -	Tics, aggression, lower threshold arousal	No / -	Face and limb dysmorphisms including prominent forehead, high anterior hairline, long smooth philtrum and thin upper lip, small anteverted nose, prominent blue iris with long eyelashes, R esotropia and brachydactyly
Fusco, WBS160	F/14.6 year	-; Severe	Anxiety, obsessive behavior	Microcephaly / Normal	Strabismus, characteristic WBS facial abnormalities, small teeth with dental malocclusion, scoliosis
Mefford, T964	F/20 year	-; Severe	-	- / Normal (age 11 y 11 mo)	Small ventricular septal defect
Röthlisberger		-; Developmental delay, including gross motor	-	Microcephaly / -	Facial dysmorphisms (bitemporal narrowing, periorbital fullness, downslanting palpebral fissures, upturned and small nose, long philtrum, full cheeks, and full lips), ventricular/atrial septal defect, minor supravalvular stenosis, anal atresia, bilateral radio-ulnar synostosis, inguinal hernia, hypothyroidism
Ramocki, 1	F/1 months	Born at 34 weeks of gestational age, death in neonatal period	-	- / Cystic encephalomalacia	—
Ramocki, 1′s father	M/34 year	-; Normal cognition	-	-	—
Ramocki, 3	M/4 year	-; GDD	ASD	- / Chiari 1	—
Ramocki, 4	F/13 year	-; Severe	-	- / -	—
Ramocki, 4′s brother	M/14 year	-; Severe	Aggressive, hyperactivity	- / -	—
Ramocki, 6	F/4 year	-; Moderate	Hyperactivity	- / -	—
Ramocki, 7	F/9 year	-; Learning disabilities	—	- / -	—
Ramocki, 7′s sister	F/11 year	-; Learning disabilities	—	- / -	—
Ramocki, 7′s father	M/38 year	-; Struggled in math	—	- / -	—
Ramocki, 8	F/13 year	-; Mild	ADHD, aggressivity, depression/mood disorder	- / -	—
Ramocki, 9	M/3 year	-; Normal cognition	—	- / -	-
Ramocki, 10	M/19 year	-; Severe/moderate	—	- / -	—
Komoike, 2	F/-	-; -	—	No / Atrophy (age 1 y 8 mo)	Facial features characteristic of WBS, supravalvular aortic stenosis
Marshall, 10	M/-	-; Severe	—	- / -	—
Marshall, 11	F/−	-; Severe	—	- / -	—
Marshall, 12	M/−	-; Severe	—	- / -	—
Marshall, 13	M/−	-; Severe, nonverbal	—	- / -	Wolff-Parkinson-White syndrome
Marshall, 14	F/−	-; Severe	—	- / -	Contractures, hypoglycemia
Marshall, 15	M/−	-; Severe	—	- / -	—
Marshall, 16	M/−	-; Severe	−	- / -	—
Marshall, 17	M/−	-; Severe	—	- / -	—
Marshall, 19	F/−	-; Severe, minimal speech	—	Macrocephaly / -	—
Marshall, 20	F/−	Minimal development	—	- / -	Blindness
Marshall, 21	M/−	-; Severe, nonverbal, cerebral palsy	—	- / -	Optic nerve hypoplasia
Marshall, 22	M/−	-; Developmental delay, nonverbal	—	- / -	—
Wu	F/6.5 year	-; Severe	Diminished interest in social interactions, self-injurious behavior, intermittent “stereotyped behavior”, sleep disturbance	Macrocephaly / -	Typical WBS features, as well as retinal abnormalities
Mizugishi	M/4 year	-; Severe, nonverbal	—	No / Mild cortical atrophy on computed tomography	Typical WBS features

Abbreviations: ADHD = attention deficit hyperactivity disorder; ASD = autism spectrum disorder; GDD = global developmental delay; ID = intellectual disability; N = no; R = right; WBS = Williams-Beuren syndrome.

Legend:.

-Not available or not applicable.

A combination of ethosuximide, valproic acid and lamotrigine was initially necessary to achieve seizure control. After discontinuation of ethosuximide, seizures remained well controlled. Subsequent discontinuation of valproic acid resulted in seizure recurrence. Other trials included 1) levetiracetam, which was ineffective, however was stopped before reaching a maximal therapeutic dose due to suspected behavioral adverse effects that subsequently did not resolve with discontinuation, and 2) topiramate, which was ineffective and had cognitive adverse effects.

He had moderate intellectual disability on formal neuropsychological testing. While early milestones were acquired on time (walked at 12 months, and first word at 11 months), he had fine motor delay, learning difficulties, and was in a specialized language class. He had behavioral issues from an early age, including aggressivity, and was later diagnosed with attention deficit hyperactivity disorder (ADHD). Treatment trials included amphetamine and methylphenidate. He also had anxiety, vocal tics and difficulty falling asleep.

His neurological examination was overall unremarkable. He had subtle dysmorphic features including dolichocephaly, low-set ears and telecanthus, however his biological father was not available for comparison. He was normocephalic and brain MRI at age five was normal. A 343-gene epilepsy panel (Fulgent Diagnostics; Temple City, California) did not reveal any pathogenic variants.

### Patient B

An 8-year-old boy, the maternal half-brother of patient A, had genetic generalized epilepsy and global developmental impairment (Tables 1 and 2). Seizures began at 2.5 years of age, and included absence and myoclonic events. Typical myoclonic seizures were captured on EEG, and corresponded to generalized polyspike-wave discharges. Hyperventilation enhanced generalized epileptiform activity, and also induced right parietal spike-wave discharges without clinical correlation. The background tracing was otherwise normal. Valproic acid resulted in at least a reduction in seizure frequency, but the patient was lost to follow-up while awaiting a repeat EEG to rule out ongoing absence seizures.

He had global developmental impairment and attended a specialized language class. Early milestones were acquired late (sat at 9 months, walked and first word at 16 to 18 months). He was diagnosed with ADHD and treated with methylphenidate. He had no other notable behavioral issues.

His neurological examination was overall unremarkable. He was normocephalic and had no definite dysmorphic features. He had no other medical diagnoses other than bilateral mild to moderate conductive hearing loss with bilateral middle ear effusions and ear tube insertions. He did not have further genetic testing beyond the chromosomal microarray analysis.

### Patient C

A 4-year-old boy, the brother of patient B, had global developmental delay (Tables 1 and 2). In infancy he developed paroxysmal episodes of unresponsiveness and staring suspicious for seizures; however, an EEG at age 2 years was normal. At the time of the last medical visit at age 2 years 10 months, he was still having staring spells, but the events appeared unlikely to be seizures.

From a developmental perspective, his early milestones were acquired on time (sat at 6 months, walked at 12 months, and first word at 9 months) but he subsequently was found to have mild-moderate global developmental delay. The fine motor and language skills were most noticeably affected. He also had oppositional behavior, as well as poor sleep, awakening 5-6 times each night.

His neurological examination was overall unremarkable, other than an intermittent, alternating fair to moderate exotropia. He also had a tendency to walk on his toes. He was normocephalic and had no dysmorphic features. He did not have further genetic testing beyond the chromosomal microarray analysis.

## Discussion

We describe a family in which multiple members carry a 7q11.23 microdeletion, distal to the common Williams-Beuren syndrome locus. Although the proband's mother had no history of seizures, her three sons all exhibited a more consistent phenotype of developmental impairment/intellectual disability and neurobehavioral abnormalities, with two of the boys having genetic generalized epilepsy. The youngest boy may yet develop a similar form of epilepsy as well, as he was last seen at only 2 years, 10 months of age. These findings demonstrate the potential for intrafamilial variability with incomplete penetrance, and also help clarify the typical epilepsy phenotype. Nevertheless, despite the similar developmental phenotype, the presence of subtle dysmorphic features in patient A, but no definite dysmorphic features in patients B and C, raises the possibility of an additional genetic “hit” inherited from a different father. Additionally, de novo mutations remain a possibility, although unlikely, in the context of further testing not being possible.

We reviewed the literature of previously reported patients with copy number deletions overlapping our patients’ coordinates (Figure 2, Table 1). We performed a search on Pubmed for “7q11 deletion”, as well as multiple searches for the genes included in our patients’ deletion (eg “HIP1”). We also reviewed manuscripts identified in the reference lists of the papers from the initial search. We have summarized the epilepsy phenotypes in these patients (Figure 2, Table 1), and also described the developmental and dysmorphic phenotype (Table 2). 34 patients were identified.^1,2,5‐15^ A large majority of these patients also had developmental impairment and/or intellectual disability, and some had reported neurobehavioral abnormalities or dysmorphic features. Most deletions included *HIP1* and *YWHAG*, with the exception of patient #4 and patient #9 described by Ramocki et al. Most patients had considerably larger deletions extending to the centromeric and/or telomeric sides, which could include the region responsible for the Williams-Beuren syndrome and 2 other candidate genes for a phenotype of epilepsy and neurodevelopmental abnormalities, *KIAA0442* and *MAGI2*.^5,10,12,15^

Williams-Beuren syndrome is a multisystem disorder caused by a heterozygous deletion on chromosome 7q11.23 not typically including *HIP1* and *YWHAG*, characterized by distinctive facial features, supravalvular aortic stenosis and mild-to-moderate intellectual disability.^6^ Seizures are rarely reported with the common deletion, but are more often described in patients with larger atypical deletions including *HIP1* and *YWHAG*, as well as *MAGI2,* who present with more pronounced neurological features than typically seen in Williams-Beuren syndrome due to common deletions.^2,5,12,16^

Patients previously reported with distal 7q11.23 deletions and epilepsy have had variable epilepsy phenotypes, including early-onset developmental and epileptic encephalopathies such as West syndrome. Later-onset epilepsy syndromes have also been described, although the latest age of seizure onset was 4 years (Table 1). Epilepsy syndromes reported have included focal and generalized syndromes, and accordingly, various focal and generalized seizure types, including infantile spasms, absence, atypical absence, myoclonic, tonic and generalized tonic-clonic seizures. Reported response to anti-seizure medications is also variable, with rapid seizure control with agents such as levetiracetam in some patients, although most described patients had drug-resistant epilepsy.

Neurodevelopmental phenotypes reported are also variable, with some patients having normal cognition or mild developmental impairment; however, most patients had more severe phenotypes including severe intellectual disability. Neurobehavioral abnormalities were described in many patients, and included ADHD, oppositional behavior and aggression. Excluding patients with Williams-Beuren syndrome and deletions in the critical 7q11.23 region, several patients have been described with dysmorphic features.

In patients with distal 7q11.23 deletions, there was variable intrafamilial penetrance. Family members of probands presenting with epilepsy, intellectual disability and neurodevelopmental abnormalities did not invariably present with all three disorders. In a study including 26 individuals from ten unrelated families with distal 7q11.23 heterozygous deletions of variable size (ranging from ∼4 Mb to ∼180 kb) including *HIP1*, Ramocki et *al* reported that probands exhibited an incomplete penetrance of epilepsy (80% of probands), neurodevelopmental disorders (58%), and learning disabilities alone (17%).^1^ For example, some individuals in the families with distal 7q11.23 deletions had normal cognition despite presenting with epilepsy. Lugo et *al* described six patients with atypical deletions in Williams-Beuren syndrome, covering multiple potentially epilepsy-related genes including *HIP1,* of which only one had a history of epilepsy (infantile spasms and other seizure types).^16^ The five other patients with at least a partial *HIP1* deletion had no history of seizures. Similarly, Marshall et *al* described four patients with Williams-Beuren syndrome and severe intellectual disability, without epilepsy.^12^

Heterozygous deletion of *MAGI2*, which was not affected in our patients, has been associated with infantile spasms.^12^ Eleven out of 21 patients with deletions including *MAGI2* had infantile spasms, excluding one patient whose seizure type was not specified (Table 1 and Supplemental Table). However, the specific importance of *MAGI2* haploinsufficiency is unclear, as these patients’ deletions also included *HIP1* and *YWHAG*. In addition, three out of 14 patients with deletions including *HIP1* and *YWHAG*, but not *MAGI2*, had infantile spasms, excluding one patient whose seizure type was not specified (Table 1 and Supplemental Table).

The role of *HIP1* in neurological disease is supported by several functional studies. Huntington-interacting-protein-1 colocalizes in hippocampal and cortical neurons with N-methyl-D-aspartate (NMDA) and a-amino-3-hydroxy-5-methyl-4-isoxazolepropionic acid (AMPA) receptors, and modulates their trafficking.^1,17,18^ NMDA and AMPA receptors play important roles in synaptic plasticity and excitotoxic cell death.^17^ Mouse models with targeted null mutation in the *HIP1* gene developed abnormal AMPA receptor trafficking and an abnormal neurological phenotype including epilepsy and failure to thrive.^1,18^

*YWHAG* encodes 14-3-3 protein gamma, a member of the 14-3-3 protein family which plays an important role in cell cycle progression, cortical development and neuronal migration. When zebrafish ywhag1 was knocked down, reduced brain size and increased diameter of the heart tube were observed, indicating that the infantile spasms and cardiomegaly seen in a patient described by Komoike et *al* may be the result of *YWHAG* haploinsufficiency.^11^

The clinical significance of heterozygous deletion of *MDH2*, which was deleted in our patients, is unclear. *MDH2* is a nuclear gene located between *HIP1* and *YWHAG*, and encodes the mitochondrial malate dehydrogenase (MDH) protein, which is essential for the conversion of malate to oxaloacetate as part of the proper functioning of the Krebs cycle.^19,20^ Ait-El-Mkadem et *al* reported bi-allelic pathogenic mutations in *MDH2* in three unrelated subjects presenting with drug-resistant epilepsy and psychomotor delay.^20^ Functional studies in fibroblasts from patients showed a loss of MDH2 protein levels and almost undetectable MDH2 enzymatic activity.^20^

Our patients’ distal 7q11.23 deletion is considerably smaller than most others reported in the literature, so the clinical description of our patients sheds light on the relative importance of the specific genes affected. Our findings, considered in combination with previous genotype-phenotype correlation and functional studies, support the hypothesis that haploinsufficiency of *HIP1* or *YWHAG* leads to a neurodevelopmental presentation that may have considerable intrafamilial phenotypic variability. In addition, there is considerable phenotypic variability between families with different deletion sizes, which could also be related to differences in which genes are included in the deletion. For example, our patients with epilepsy all had genetic generalized epilepsy; however, other patients with 7q11.23 deletions of smaller or larger size have been reported with focal or other generalized epilepsy syndromes, including epileptic encephalopathies.. Better understanding of the roles of *HIP1* and *YWHAG* may eventually offer avenues for rational drug design, including antiseizure agents, in these patients who often present with drug-resistant epilepsy.^5^

## Supplemental Material

sj-docx-1-cno-10.1177_2329048X221093173 - Supplemental material for Genetic Generalized Epilepsy and Intrafamilial Phenotypic Variability with Distal 7q11.23 Deletion Click here for additional data file. Supplemental material, sj-docx-1-cno-10.1177_2329048X221093173 for Genetic Generalized Epilepsy and Intrafamilial Phenotypic Variability with Distal 7q11.23 Deletion by Veronica Birca and Kenneth A. Myers in Child Neurology Open
