# Supplementary material for: Genetic Generalized Epilepsy and Intrafamilial Phenotypic Variability with Distal 7q11.23 Deletion
Source: Child Neurol Open. 2022 Apr 21;9:2329048X221093173. doi: 10.1177/2329048X221093173 (PMC9036355; doi:10.1177/2329048X221093173)
Supplement: sj-docx-1-cno-10.1177_2329048X221093173 - Supplemental material for Genetic Generalized Epilepsy and Intrafamilial Phenotypic Variability with Distal 7q11.23 Deletion [file sj-docx-1-cno-10.1177_2329048X221093173.docx]

**Supplementary Table. Deletion Breakpoints in Patients With Distal 7q11.23 Deletions and Epilepsy**

| **Publication author, patient ID** | **Deletion size**** | **Breakpoints remapped to hg19 [Original breakpoints] (build)***** | **Candidate genes****** |
| --- | --- | --- | --- |
| A (current publication) | 0.88 Mb | 75163662-76047662 (hg19) | *HIP1, MDH2, YWHAG* |
| B (current publication) | 0.88 Mb | 75163662-76047662 (hg19) | *HIP1, MDH2, YWHAG* |
| C (current publication) | 0.88 Mb | 75163662-76047662 (hg19) | *HIP1, MDH2, YWHAG* |
| Nicotera | 19.4 Mb | 66849415-86269865 (-) | *KIAA0442, HIP1, MDH2, YWHAG, MAGI2* |
| Nicita, P1 | 1.92 Mb | 75061956-76989532 (hg19) | *HIP1, MDH2, YWHAG* |
| Epi4K Consortium, cy | 11.4 Mb | 75157776-86595291 (hg19) | *HIP1, MDH2, YWHAG, MAGI2* |
| Peterson, - | 5.1 Mb | 75165711-80260623 (hg19)  [75003647-80098559] (hg18) | *HIP1, MDH2, YWHAG, MAGI2* |
| Fusco, WBS160 | 4.22 Mb | 72473032-76696036 (hg19) | *HIP1, MDH2, YWHAG* |
| Mefford, T964 | 8 Mb | 74142064-82142064 (hg19)  [73780000-81980000] (hg18) | *HIP1, MDH2, YWHAG, MAGI2* |
| Röthlisberger | 13.2 Mb | 63262565-76482064 (hg19)  [62900000-76320000] (hg18) | *KIAA0442, HIP1, MDH2, YWHAG* |
| Ramocki, 1 | 1.12 Mb | *75162064-*76282064 (hg19)  [*75000000-*76120000] (hg18) | *HIP1, MDH2, YWHAG* |
| Ramocki, 1’s father | 1.12 Mb | *75162064-*76282064 (hg19)  [*75000000-*76120000] (hg18) | *HIP1, MDH2, YWHAG* |
| Ramocki, 3 | 1.12 Mb | *75162064-*76282064 (hg19)  [*75000000-*76120000] (hg18) | *HIP1, MDH2, YWHAG* |
| Ramocki, 4 | 0.19 Mb | *75162064-*75352064 (hg19)  [*75000000-*75190000] (hg18) | *HIP1* |
| Ramocki, 4’s brother | 0.19 Mb | *75162064-*75352064 (hg19)  [*75000000-*75190000] (hg18) | *HIP1* |
| Ramocki, 6 | 1.12 Mb | *75162064-*76282064 (hg19)  [*75000000-*76120000] (hg18) | *HIP1, MDH2, YWHAG* |
| Ramocki, 7 | 1.12 Mb | *75162064-*76282064 (hg19)  [*75000000-*76120000] (hg18) | *HIP1, MDH2, YWHAG* |
| Ramocki, 7’s sister | 1.12 Mb | *75162064-*76282064 (hg19)  [*75000000-*76120000] (hg18) | *HIP1, MDH2, YWHAG* |
| Ramocki, 7’s father | 1.12 Mb | *75162064-*76282064 (hg19)  [*75000000-*76120000] (hg18) | *HIP1, MDH2, YWHAG* |
| Ramocki, 8 | 1.12 Mb | *75162064-*76282064 (hg19)  [*75000000-*76120000] (hg18) | *HIP1, MDH2, YWHAG* |
| Ramocki, 9 | 0.28 Mb | *75162064-*75442064 (hg19)  [*75000000-*75280000] (hg18) | *HIP1* |
| Ramocki, 10 | 1.12 Mb | *75162064-*76282064 (hg19)  [*75000000-*76120000] (hg18) | *HIP1, MDH2, YWHAG* |
| Komoike, 2 | 3.9 Mb | 72700414-76637472 (hg19)  [72338350-76475408] (hg18) | *HIP1, MDH2, YWHAG* |
| Marshall, 10 | 4.4 Mb | *72250000-*76400000 (-) | *HIP1, MDH2, YWHAG* |
| Marshall, 11 | 6.7 Mb | *70850000/71250000-*77500000 (-) | *HIP1, MDH2, YWHAG* |
| Marshall, 12 | 5.5 Mb | *72250000-*77800000 (-) | *HIP1, MDH2, YWHAG, MAGI2* |
| Marshall, 13 | 11-12.5 Mb | *70500000-*78400000/79000000 (-) | *HIP1, MDH2, YWHAG, MAGI2* |
| Marshall, 14 | 10 Mb | *70500000-*79000000 (-) | *HIP1, MDH2, YWHAG, MAGI2* |
| Marshall, 15 | 8.3 Mb | *72250000-*80350000 (-) | *HIP1, MDH2, YWHAG, MAGI2* |
| Marshall, 16 | 11 Mb | *70500000-*80500000 (-) | *HIP1, MDH2, YWHAG, MAGI2* |
| Marshall, 17 | 17 Mb | *70500000-*82900000 (-) | *HIP1, MDH2, YWHAG, MAGI2* |
| Marshall, 19 | >9 Mb | *72250000-*82000000/86200000 (-) | *HIP1, MDH2, YWHAG, MAGI2* |
| Marshall, 20 | 17 Mb | *71500000-*86200000 (-) | *HIP1, MDH2, YWHAG, MAGI2* |
| Marshall, 21 | 26 Mb | *70500000-*86200000 (-) | *HIP1, MDH2, YWHAG, MAGI2* |
| Marshall, 22 | 11.5 Mb | *74850000-*86200000 (-) | *HIP1, MDH2, YWHAG, MAGI2* |
| Wu | >9.3 Mb | *72744454-*>82073272 (-) | *HIP1, MDH2, YWHAG* |
| Mizugishi | 12.5 Mb | *74126556-*77674081 (-) | *HIP1, MDH2, YWHAG, MAGI2* |

Abbreviations: mo = month(s); y = years; hg = human genome.

Legend:

-Not available or not applicable.

*CNV breakpoint estimated from figures in original publications, or provided information on deleted genes, since precise coordinates were not given by the authors.

**Deletion size after genome remapping, where applicable

***The slash symbol "/" indicates a range of breakpoint, since the precise breakpoint was undefined in the original publications.

****Genes associated with epilepsy and developmental delay

>Extending beyond
